# Psychological and educational learning strategies and models during the COVID-19 pandemic: A comparative bibliometric analysis

**DOI:** 10.3389/fpsyg.2022.1029812

**Published:** 2022-11-21

**Authors:** Abdullah Falah M. Alshahrani

**Affiliations:** Department of Education, University of Bisha, Bisha, Saudi Arabia

**Keywords:** bibliometric analysis, psychological and educational learning strategies, VOSviewer, models, COVID-19

## Abstract

This paper undertakes a literature review of psychological, Educational Learning Strategies, and Models during the COVID-19 Pandemic. It examines data from 359 publications relating to this subject, published on the Web of Science, Scopus, and ScienceDirect between 2020 and 2021 using bibliometric analysis adapted with VOSviewer software. The review discusses the following approaches (keywords, authors, references (research papers), research work, countries, and research institutions). It concluded that bibliometric analysis is fundamental for detailing the theoretical literature and developing an integrated theoretical framework for psychological and Educational Learning Strategies. The psychological impact on students and potential stress needs to be closely monitored and evaluated, to plan effective policies while adopting these pedagogical approaches.

## Introduction

Due to the significant movement to distance education that has occurred in response to the Coronavirus Disease 2019 (COVID-19) pandemic. As almost all teaching methods have rapidly moved to distance education, it is now imperative that consistent distance education approaches be developed using psychological and pedagogical learning methodologies (e.g., Dede,1996; Bates, 2008; Chang and Huang, 2012; Adedoyin and Soykan, 2020; Ali, 2020; Johnson et al., 2020; Mahyoob, 2020).

If, as Astley suggests, the wide variety of research projects is due to the many subfields of study that make up the educational sciences (Astley 2019), the process of preparing research in various fields is intricately tied to several frameworks. The work that has come before is tedious and repetitious, and there is so much of it that it may be hard for a researcher to define ideas, plot a route for their study, or even realize that they have gotten off track because they are not familiar with the work of prominent scholars. Who can rely on, control, and deal with this large body of this research?

Some databases organize research (like Web of Science, ISI, Scopus, and Google Scholars). As management sciences and researchers prepare increasing numbers of distinguished studies, there is a need to explore how to manage this increase in literature.

Computer software helps manage data, enabling it to be organized, stored, published, and distributed. Through such resources, we are able to process increasing numbers of previous studies. For example, software such as Citespac and VOSviewer help us know the most influential researchers globally. Therefore, the field should focus on references, keywords, research cases, and organizations.

This study undertook bibliometric analysis, examining and comparing previous methods (including meta-analysis and systematic reviews), with a focus on psychological, Educational Learning Strategies during COVID-19. This paper analyzed the production of psychological educational learning strategies as indexed in Web of Science (WOS) and Scopus (2020–2021). According to Mulet Bayona et al. (2021), the research questions of this study are:

RQ. What does bibliometric analysis contribute to the review and development of the theoretical literature on psychological and Educational Learning Strategies?

The sub-questions are:

RQ1. What role does bibliometric analysis play in establishing the PELS theoretical frameworks?

RQ2. What structure is produced by the publications and citations that constitute PELS quality?

RQ3. Which keywords do PELS authors use most frequently?

RQ4. Who are the most frequently mentioned writers in the area of PELS?

RQ5. What are the most frequently cited research publications in the area of PELS?

RQ6. What are the most significant research institutes for producing research papers in PELS?

RQ7. Which countries contribute the most to the development of research publications in PELS? (Q5 1 R2).

This research will enable us to discover research trends in this field and help familiarize readers with the topic and become more knowledgeable about the development of Psychological and Educational Learning Strategies in the scientific community. Likewise, the justification and significance of the analysis in this study are based on seven research questions that guided the work. The major objective is to identify the source titles, institutions, authors, and countries with the largest scientific output on higher education, as well as to identify higher education trends in the scientific literature.

The purpose of the study is to determine the results and process of bibliometric analysis, which will aid the researcher in educational sciences in finding distinctions for research, including identifying keywords, the most influential researchers in the field, the research work, reference sources, countries, and reference research institutions. Consequently, the research compares bibliometric analysis to conventional literature evaluations in the educational sciences, considering of bibliometric analysis and research techniques for bibliometric studies in the educational sciences. In addition to a bibliographic examination of the psychological problems faced by students during the COVID-19 pandemic, this study includes a discussion of its significance.

The deductive hypothesis methodology (hypothetical-deductive method) was adopted, which begins by examining the initial assumptions (hypotheses) and trying to support or test them according to what theories for bibliometric analysis have been developed previously concerning psychological and Educational Learning Strategies.

## Materials and methods

Bibliometric analysis is a method of science mapping that analyzes data from previous research and studies, considering how articles, authors, areas and resources are related to one another (five methods include Citation, Co-citation, Bibliographic coupling, Co-author, and Co-word), defining the critical areas of research, keywords, authors, countries, and organizations in a specific field of knowledge (Chang and Huang, 2012; Zupic and Čater, 2015; Hallinger and Chatpinyakoop, 2019; Su et al., 2020) such as E-learning strategies in COVID-19.

To carry out the study, data were taken from three approved databases, namely Web of Science (ISI), Scopus, and Science Direct. We retrieved published research *via* a topic search of the psychological and Educational Learning strategies and models used during the COVID-19 Pandemic using the WoS database on January 5, 2020. The following search terms were used: topic = (“the psychological and Educational Learning strategies” “COVID-19” “models” “Pandemic“), in title-abs-key from 2020 to 2021. There were 359 studies distributed over 2 years.

### Web of science (ISI) data

**Figure 1:**
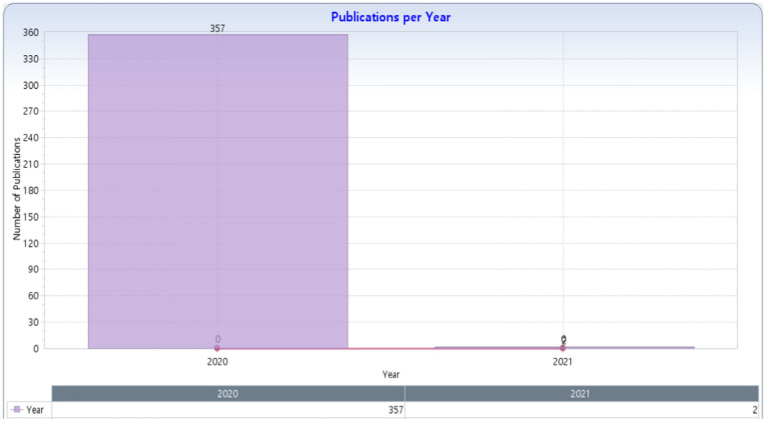
Publications per year in Web of Science (KnowledgeMatrix Plus outputs).

The 359 studies retrieved included articles, conferences, and book chapters, as shown in Figure 1.

Figure 1 shows the number of studies used in the bibliometric analysis, distributed as follows: 357 studies in 2020 and 2 studies in 2021, with a total of 359 studies.

### Scopus data

**Figure 2:**
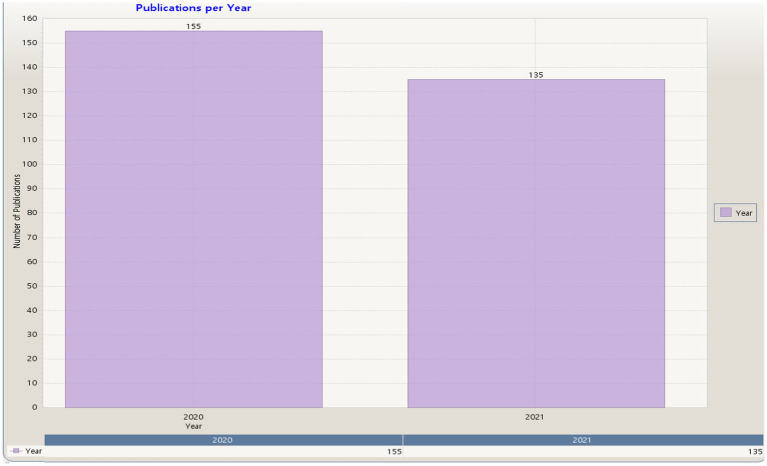
Publications per year in Scopus (KnowledgeMatrix Plus outputs).

In total, we retrieved 290 studies on the topic of learning strategies and models in COVID-19 from the Scopus database, as shown in Figure 2.

Figure 2 shows the number of studies used in the bibliometric analysis of the Scopus database including, 155 studies in 2020 and 135 studies in 2021, with a total of 290 studies.

### Science direct data

**Figure 3:**
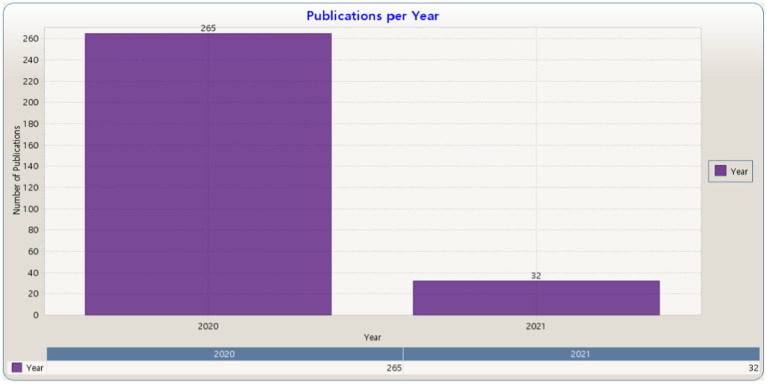
Publications per year in Science Direct (KnowledgeMatrix Plus outputs).

Figure 3 presents the distribution of 297 studies on the topic of learning strategies and models in COVID-19 retrieved from the Science Direct database.

Figure 3 shows the number of studies used in the bibliometric analysis relying on the Science Direct database, including 265 studies in 2020 and 32 studies in 2021, with a total of 297 studies.

### Analysis approaches

To prepare the bibliometric analysis, the number of publications and citations were used to find the relationship between keywords, based on outputs of two software KnowledgeMatrix Plus KISTI (2016) and VOSviewer (Van Eck and Waltman, 2017; Van Eck and Waltman, 2020).

## Results of study

### Web of science data

**Figure 4:**
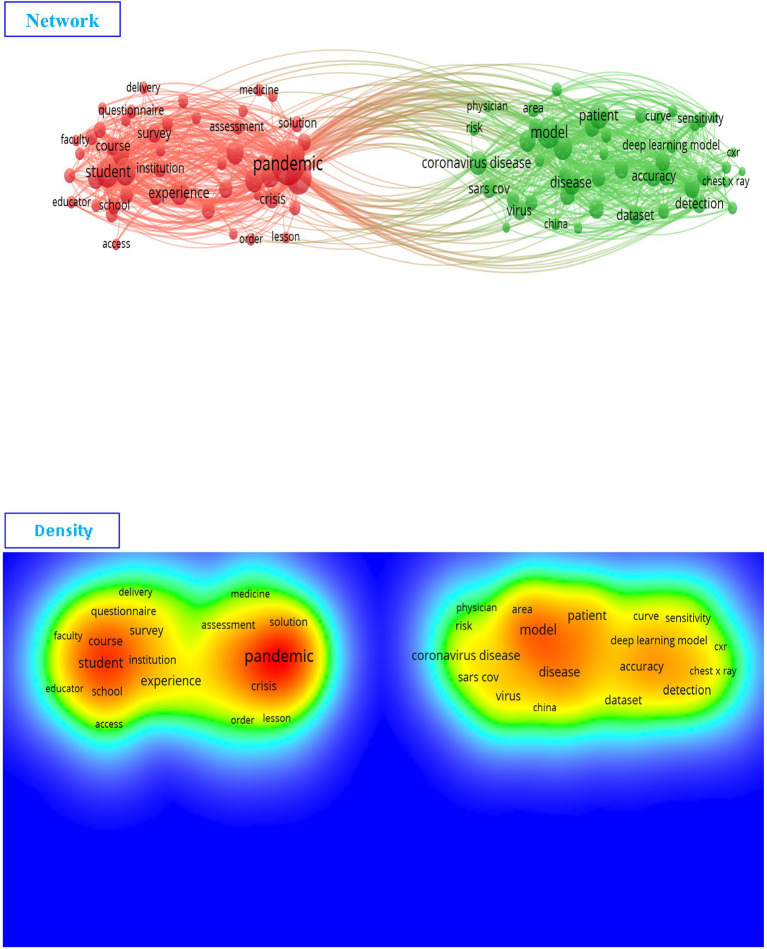
The network and density of keywords depend on Web of Science (VOSviewer outputs).

Figure 4 shows the results of a bibliometric analysis (network and density) for keywords in learning strategies and models used during COVID-19 (Appendix 1).

In terms of network and density, Figure 4 shows that there are two sub-fields of research on the subject of learning strategies and models in COVID-19. The first field (the red cluster) is mainly related to the pandemic, student, and course, etc. (Appendix 1). The second field (the green cluster) is related to the model, deep learning, and COVID-19, etc. For more details, the Table 1 shows the keywords missed within each cluster.

**Table 1:** Learning strategies and models in COVID-19 depending on Web of Science (VOSviewer outputs).

Clusters	Keywords
Cluster 1 (50 items)	Access, assessment, challenge, communication, community, context, course, crisis, delivery, distance, distance learning, e-learning, education, educator, environment, era, experience, face, faculty, goal, home, impact, institution, learning, lesson, medicine, online, online learning, opportunity, order, pandemic, participant, perspective, platform, quality, questionnaire, recommendation, response, school, situation, social distancing, solution, student, survey, teacher, teaching, technology, transition, university.
Cluster 2 (47 items)	Accuracy, algorithm, area, artificial intelligence, AUC, case, chest x-ray, chest x-ray image, china, classification, CNN, convolutional neural network, coronavirus, coronavirus disease, curve, cxr, dataset, death, deep learning, deep learning model, detection, diagnosis, disease, feature, hospital, identification, image, individual, infection, machine, machine learning, model, patient, performance, physician, pneumonia, prediction, radiologist, risk, sars cov, sensitivity, specificity, technique, testing, treatment, vaccine, virus.

Table 1 shows that are two approaches to research on the topic of learning strategies and models in light of the COVID-19 pandemic, the first related to research on the learning strategies and curricula used in COVID-19, such as e-learning, distance learning, online learning, platform, quality, school, student, teacher, teaching, technology, transition, and university (the first cluster), similar with the results of studies (Ali, 2020; Mahyoob, 2020; Mpungose, 2020; Elumalai et al., 2021). The second is related to machine learning and deep learning, which has been widely talked about during the pandemic, such as artificial intelligence, chest x-ray image, convolutional neural network, coronavirus disease deep learning, diagnosis, machine learning, patient, radiologist, SARS CoV, testing, treatment, vaccine, and virus (the second cluster), showing results that are similar to those of another study (Fusco et al., 2021). This confirms the different research strategies on this topic, such as the different learning strategies and models. In addition, there are two completely different directions of research on this topic.

### Scopus data

The next figure shows the results of the bibliometric analysis of keywords using the Scopus database.

**Figure 5:**
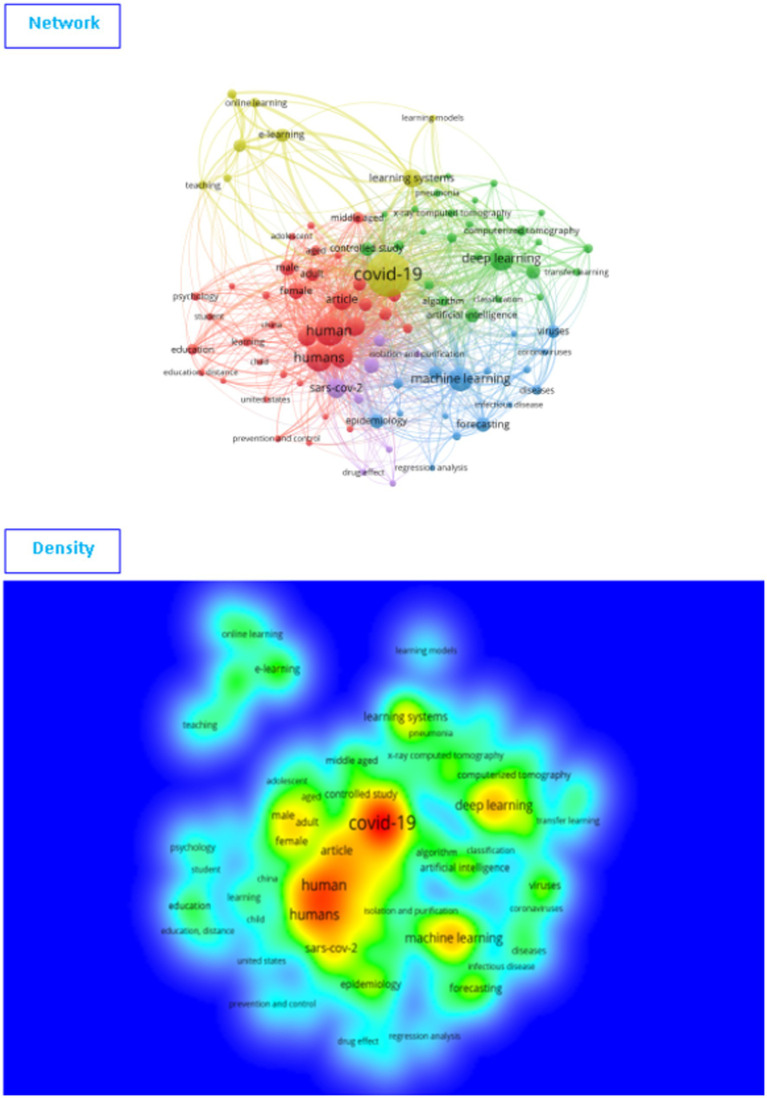
The network and density of keywords depend on Scopus (VOSviewer outputs).

Figure 5 shows that there are five sub-fields of research on the subject of learning strategies and models used during the COVID-19 pandemic. The first field (the red cluster) mainly related to human, adult, gender. The second field (the yellow cluster) is mainly related to COVID-19, learning systems, online learning, and e-learning. The third field (the green cluster) relates to deep learning, and artificial intelligence. The fourth field (the blue cluster) is related to machine learning, forecasting, and the fifth field (the purple cluster) is related to SARS CoV-2, isolation and purification (Appendix 2), as shown in Table 2.

**Table 2:** Learning strategies and models in COVID-19 depending on Scopus (VOSviewer outputs).

Clusters	Keywords
Cluster 1 (36 items)	Adolescent, adult, aged, betacoronavirus, child, China, computer simulation, coronavirus disease, coronavirus infection, coronavirus infections, education, distance, epidemic, female, global health, human, humans, learning, male, medical education, middle-aged, models, pandemic, pandemics, pneumonia, prevention, psychology, retrospective, risk factor, student, theoretical model, United State, viral, virus pneumonia, young adult.
Cluster 2 (26 items)	Algorithm, algorithms, artificial intelligence, biological organs, classification, information, computerized, controlled study, CNN, conventional neural network, deep learning, diagnosis, diagnostic imaging, diagnostic test accuracy, feature extraction, image processing, image segmentation, lung, major clinical study, pneumonia, receiver operating, sensitivity, thorax radiology, tomography, transfer learning, x-ray computed tomography.
Cluster 3 (16 items)	Coronavirus, coronavirus, decision making, decision trees, diseases, epidemiology, forecasting, infectious disease, learning algorithms, machine learning, ML mode, prediction, predictive analytics, public health, regression analysis, viruses.
Cluster 4 (9 items)	COVID-19, e-learning, education computing, higher education, learning models, learning systems, online learning, students, teaching.
Cluster 5 (8 items)	Drug effect, drug repositioning, isolation, priority journal, procedures, sars-cov-2, severe acute respiratory, virology.

Table 2 shows that there are five research directions or approaches that the researcher can take in the topic of learning strategies and models in COVID-19, but the basis for them is the existence of two real directions: the first is related to research on learning strategies and curricula used during COVID-19 (e-learning, distance learning, and online learning), which includes cluster four [see the result of study (Rahimi et al., 2021; Subramanian et al., 2022)]. The second is related to machine learning and deep learning (artificial intelligence in COVID-19). It includes clusters one, two, three, and five.

### Science direct data

**Figure 6:**
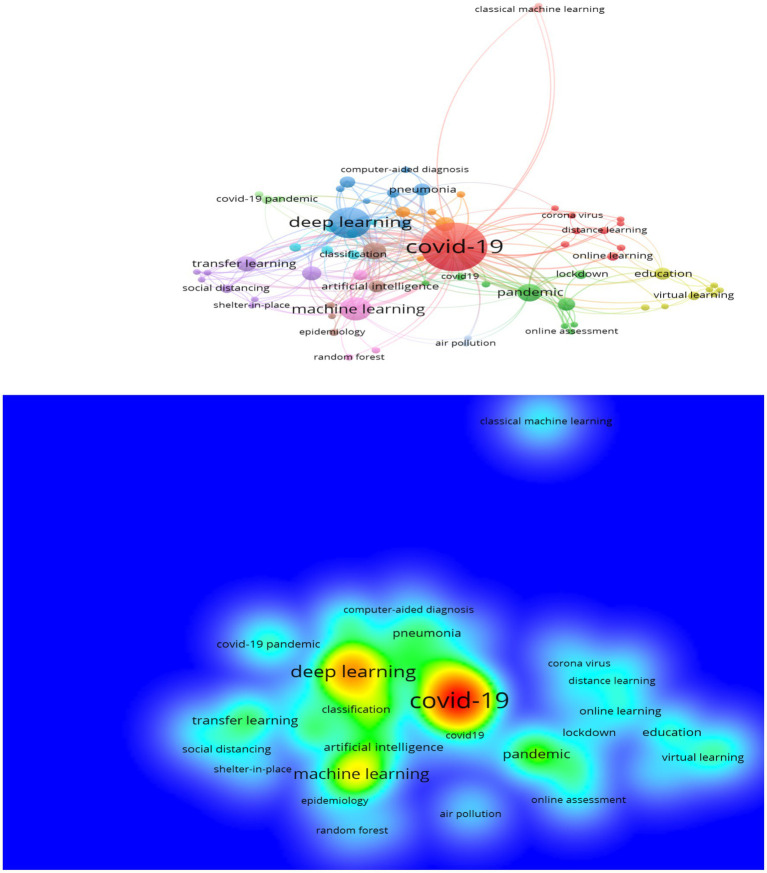
The network and density of keywords depend on Science Direct (VOSviewer outputs).

Figure 6 shows the results of the bibliometric analysis of keywords retrieved from the Science Direct database.

As Figure 6 shows, there are eight sub-fields of research on the subject of learning strategies and models in COVID-19. The first field (the red cluster) shows COVID-19 (Appendix 3). The second field (the blue cluster) shows deep learning. The third field (the green cluster) was related to the pandemic. The fourth field (the pink cluster) shows machine learning. The fifth field (the purple cluster) is related to transfer learning. The sixth field (the purple cluster) relates to education, and the seventh field (the orange cluster) shows pneumonia. The eighth field (the brown cluster) is related to artificial intelligence, as indicated in more detail in Table 3.

**Table 3:** Learning strategies and models in COVID-19 depending on Science Direct (VOSviewer outputs).

Clusters	Keywords
Cluster 1 (10 items)	Acceptance, coronavirus, COVID-19, deep learning models, distance learning, online learning, parents, perception, plastic surgery, training
Cluster 2 (9 items)	Covid19, e-learning, Jordan, lockdown, online assessment, online pbl, pandemic, sars-cov2, virtual classroom
Cluster 3 (8 items)	Bayesian optimisation, CNN, computed tomography, computer-aided diagnostics, coronavirus disease 2019, deep learning, pneumonia, x-ray
Cluster 4 (8 items)	Binary logistic regression, COVID-19 outbreak, education, medical student education, propensity model, remote learning, student’s health behaviour, virtual learning
Cluster 5 (8 items)	Overhead view, person detection, sars-cov-2, shelter-in-place, social distancing, statistics, transfer learning, yolov3
Cluster 6 (6 items)	Chest x-ray, Chest x-ray images, classification, convolutional neural network, convolutional neural networks, long short-term memory
Cluster 7 (5 items)	Forecasting, incremental learning, lstm, modelling, optimization
Cluster 8 (5 items)	Artificial intelligence, coronavirus, data science, epidemiology, topic modelling

Table 3 displays similar results to the previous tables. There are two research directions or approaches related to the topic of learning strategies and models in COVID-19. The first is related to research on the learning strategies and curricula used in COVID-19 (e-learning, distance learning, and online learning) (Chang and Fang, 2020, Kamysbayeva et al., 2021; Sharin 2021). It includes cluster one, two, and four. The second is related to machine learning and deep learning (artificial intelligence in COVID-19). It includes clusters three, five, six, seven, and eight.

## Conclusion and implementations

This review used bibliometric network analysis to provide an overview of researchers worldwide’ studies on psychological and educational learning strategies and models used all over the world during the COVID-19 pandemic. The sample included 359 articles indexed in Web of Science (ISI) data, 290 articles indexed by Scopus Data, and 297 articles indexed by Science Direct data. It provides insights into the dissemination of knowledge among researchers in this field. According to the main review findings, several topics were highlighted and may be further reviewed in the future.

The impact of the COVID-19 pandemic has been profound. It was necessary to radically change the style of teaching and learning depending on the current conditions imposed by the pandemic, including the tools and strategies used by institutions of higher education and the experiences of faculty members. This transition has had a psychological and educational impact on students, which has led to students’ anxiety and stress (see Akour et al., 2020; Ali, 2020; Mishra et al., 2020; Radu et al., 2020; Brika et al., 2021; Zalat et al., 2021; Yahiaoui et al., 2022).

There are also many important messages for the post-coronavirus period. Among them, faculty members and universities were not exposed to many required teaching methods. Also, many of them had to go through a very steep learning curve to be able to deliver basic lessons. Many studies also covered the educational impact on students and the strategies used by higher education institutions in all countries of the world. The literature on the psychological impact and the strategies used is limited to a few countries, as discussed in a study by Son et al. (2020). There is much work to be done to understand the psychological effects on students at universities in many countries, including developing ones.

COVID-19 has had a profound impact on students, and its impact on student’s mental health and well-being cannot be fully understood. Many of the current psychological assessments in this area are short-term reviews. Perhaps well-studied longitudinal studies can shed light on this in the future.

Colleges and universities have also had to deal with a lot of change during this time. However, the psychological impact on students and potential stress needs to be closely monitored and evaluated, to formulate effective policies while adopting these pedagogical approaches.

## Data availability statement

The original contributions presented in the study are included in the article/Supplementary material, further inquiries can be directed to the corresponding author.

## Author contributions

The author confirms being the sole contributor of this work and has approved it for publication.

## Funding

The author extends their appreciation to the Deputyship for Research and Innovation, Ministry of Education in Saudi Arabia for funding this research (under project number UB-36-1442).

## Conflict of interest

The author declares that the research was conducted in the absence of any commercial or financial relationships that could be construed as a potential conflict of interest.

## Publisher’s note

All claims expressed in this article are solely those of the authors and do not necessarily represent those of their affiliated organizations, or those of the publisher, the editors and the reviewers. Any product that may be evaluated in this article, or claim that may be made by its manufacturer, is not guaranteed or endorsed by the publisher.
