# Supplementary material for: Psychological and educational learning strategies and models during the COVID-19 pandemic: A comparative bibliometric analysis
Source: Front Psychol. 2022 Nov 21;13:1029812. doi: 10.3389/fpsyg.2022.1029812 (PMC9721261; doi:10.3389/fpsyg.2022.1029812)
Supplement: Supplementary file 1 [file Data_Sheet_1.doc]

***ANNEXES***

***Appendix 1.***

*The most frequent keywords in learning strategies in COVID-19 (Web of Science)*

| *Keyword* | *occurrences* | *relevance score* |
| --- | --- | --- |
| *pandemic* | *223* | *0.3434* |
| *learning* | *134* | *0.3548* |
| *model* | *112* | *0.4885* |
| *student* | *105* | *1.7372* |
| *education* | *90* | *1.6529* |
| *patient* | *82* | *0.8128* |
| *case* | *72* | *0.6088* |
| *experience* | *71* | *1.0305* |
| *impact* | *70* | *0.4979* |
| *challenge* | *69* | *0.4886* |
| *coronavirus disease* | *69* | *0.2594* |
| *disease* | *67* | *0.6446* |
| *accuracy* | *60* | *1.0814* |
| *response* | *59* | *0.5922* |
| *teaching* | *58* | *1.7793* |
| *performance* | *57* | *0.4081* |
| *university* | *56* | *1.6401* |
| *image* | *53* | *1.3507* |
| *infection* | *48* | *0.7816* |
| *machine* | *47* | *0.8146* |
| *diagnosis* | *46* | *1.147* |
| *crisis* | *45* | *0.425* |
| *detection* | *45* | *1.3019* |
| *machine learning* | *45* | *0.6983* |
| *technique* | *43* | *0.6604* |
| *virus* | *43* | *0.469* |
| *algorithm* | *42* | *0.9276* |
| *pneumonia* | *41* | *1.4851* |
| *sars cov* | *41* | *0.2998* |
| *feature* | *40* | *0.7973* |
| *coronavirus* | *39* | *0.4093* |
| *course* | *39* | *1.4364* |
| *deep learning* | *39* | *1.3153* |
| *survey* | *37* | *1.3567* |
| *technology* | *37* | *0.6031* |
| *institution* | *36* | *1.239* |
| *school* | *36* | *1.8314* |
| *area* | *34* | *0.3757* |
| *distance* | *33* | *1.7369* |
| *community* | *32* | *0.3606* |
| *face* | *32* | *1.5439* |
| *opportunity* | *32* | *1.2292* |
| *prediction* | *32* | *0.9388* |
| *sensitivity* | *31* | *1.4734* |
| *teacher* | *30* | *1.9517* |
| *online learning* | *29* | *1.9345* |
| *specificity* | *29* | *1.5701* |
| *context* | *28* | *0.3946* |
| *treatment* | *28* | *0.6082* |
| *death* | *27* | *0.7211* |
| *risk* | *26* | *0.2601* |
| *deep learning model* | *23* | *1.3288* |
| *questionnaire* | *23* | *1.6569* |
| *lesson* | *21* | *0.3413* |
| *chest x-ray image* | *20* | *1.5251* |
| *China* | *20* | *0.5694* |
| *distance learning* | *20* | *2.1375* |
| *e-learning* | *20* | *1.8241* |
| *social distancing* | *20* | *0.8454* |
| *artificial intelligence* | *19* | *0.9025* |

***Appendix 2.***

*The most frequent keywords in learning strategies in COVID-19 (Scopus)*

| *Keyword* | *occurrences* | *total link strength* |
| --- | --- | --- |
| *covid-19* | *172* | *151.00* |
| *human* | *85* | *85.00* |
| *humans* | *75* | *75.00* |
| *deep learning* | *62* | *58.00* |
| *machine learning* | *61* | *60.00* |
| *pandemic* | *59* | *57.00* |
| *article* | *46* | *46.00* |
| *coronavirus disease 2019* | *44* | *44.00* |
| *sars-cov-2* | *44* | *43.00* |
| *pandemics* | *41* | *41.00* |
| *learning systems* | *40* | *39.00* |
| *female* | *32* | *32.00* |
| *male* | *30* | *30.00* |
| *procedures* | *29* | *29.00* |
| *forecasting* | *27* | *27.00* |
| *artificial intelligence* | *26* | *26.00* |
| *diagnosis* | *25* | *25.00* |
| *epidemiology* | *25* | *25.00* |
| *adult* | *24* | *24.00* |
| *controlled study* | *24* | *24.00* |
| *e-learning* | *24* | *23.00* |
| *viruses* | *23* | *23.00* |
| *coronavirus* | *22* | *21.00* |
| *coronavirus infection* | *22* | *22.00* |
| *coronavirus infections* | *22* | *22.00* |
| *virus pneumonia* | *22* | *22.00* |
| *pneumonia, viral* | *21* | *21.00* |
| *students* | *21* | *21.00* |
| *middle-aged* | *18* | *18.00* |
| *priority journal* | *18* | *18.00* |
| *education* | *17* | *16.00* |
| *algorithm* | *16* | *16.00* |
| *computerized tomography* | *16* | *16.00* |
| *betacoronavirus* | *15* | *15.00* |
| *major clinical study* | *15* | *15.00* |
| *online learning* | *15* | *12.00* |
| *public health* | *15* | *14.00* |
| *aged* | *14* | *14.00* |
| *diagnostic imaging* | *14* | *14.00* |
| *diseases* | *14* | *14.00* |
| *algorithms* | *13* | *13.00* |
| *convolutional neural networks* | *13* | *13.00* |
| *education computing* | *13* | *13.00* |
| *virology* | *13* | *13.00* |
| *learning* | *12* | *12.00* |
| *teaching* | *12* | *12.00* |
| *decision making* | *11* | *11.00* |
| *higher education* | *11* | *10.00* |
| *image processing* | *11* | *11.00* |
| *psychology* | *11* | *11.00* |
| *x-ray computed tomography* | *11* | *11.00* |
| *China* | *10* | *10.00* |
| *decision trees* | *10* | *10.00* |
| *machine learning models* | *10* | *10.00* |
| *pneumonia* | *10* | *10.00* |
| *risk factor* | *10* | *10.00* |
| *tomography, x-ray computed* | *10* | *10.00* |
| *transfer learning* | *10* | *10.00* |
| *adolescent* | *9* | *9.00* |
| *classification* | *9* | *9.00* |
| *classification (of information)* | *9* | *9.00* |
| *convolutional neural network* | *9* | *9.00* |
| *education, distance* | *9* | *9.00* |
| *image segmentation* | *9* | *9.00* |
| *prevention and control* | *9* | *9.00* |
| *student* | *9* | *9.00* |
| *theoretical model* | *9* | *9.00* |
| *young adult* | *9* | *9.00* |
| *biological organs* | *8* | *8.00* |
| *coronaviruses* | *8* | *8.00* |
| *diagnostic test accuracy study* | *8* | *8.00* |
| *drug effect* | *8* | *8.00* |
| *drug repositioning* | *8* | *7.00* |
| *epidemic* | *8* | *8.00* |
| *feature extraction* | *8* | *8.00* |
| *learning models* | *8* | *8.00* |
| *lung* | *8* | *8.00* |
| *models, theoretical* | *8* | *8.00* |
| *prediction* | *8* | *8.00* |
| *retrospective study* | *8* | *8.00* |
| *sensitivity and specificity* | *8* | *8.00* |
| *severe acute respiratory syndrome coronavirus 2* | *8* | *8.00* |
| *child* | *7* | *7.00* |
| *computer simulation* | *7* | *7.00* |
| *global health* | *7* | *7.00* |
| *infectious disease* | *7* | *7.00* |
| *isolation and purification* | *7* | *7.00* |
| *learning algorithms* | *7* | *7.00* |
| *medical education* | *7* | *7.00* |
| *predictive analytics* | *7* | *7.00* |
| *receiver operating characteristic* | *7* | *7.00* |
| *regression analysis* | *7* | *7.00* |
| *thorax radiography* | *7* | *7.00* |
| *united states* | *7* | *7.00* |
| *viral disease* | *7* | *7.00* |

***Appendix 3.***

*The most frequent keywords in learning strategies in COVID-19 (Science Direct)*

| *Keyword* | *occurrences* |
| --- | --- |
| *covid-19*  *Deep learning*  *Machine learning*  *Pandemic*  *Coronavirus*  *Forecasting*  *Sars-cov- 02*  *Transfert learning*  *Pneumonia*  *Education*  *Computed tomography*  *Prediction*  *LSTM*  *Machine learning*  *Classification*  *CNN*  *Social distancing*  *Online learning*  *Lockdown*  *Artificial intelligence* | *177*  *78*  *24*  *21*  *18*  *17*  *15*  *13*  *12*  *11*  *10*  *10*  *8*  *8*  *8*  *7*  *7*  *7*  *7*  *6* |

***
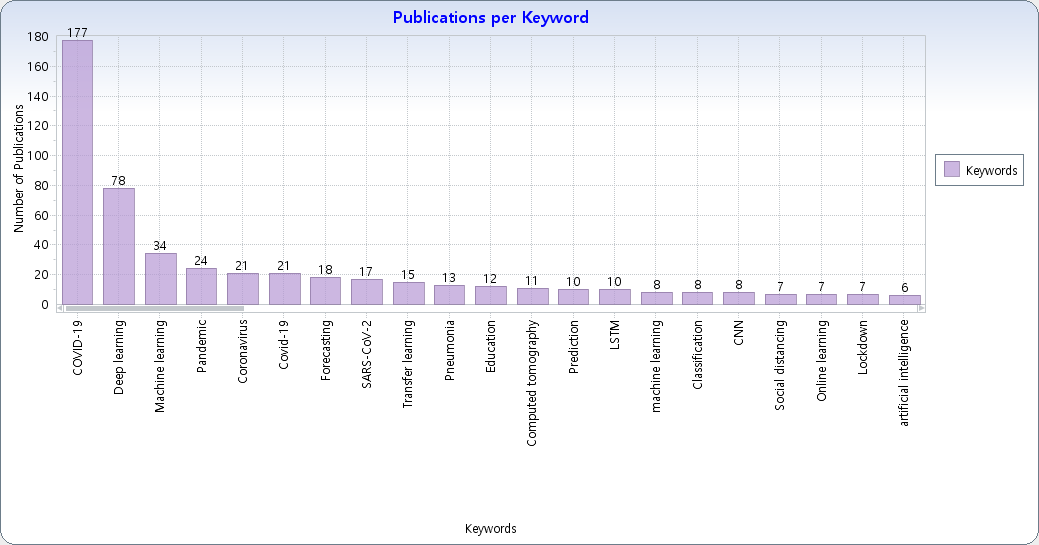
***
